# How Far Does Highly Active Antiretroviral Treatment Reduce TB Incidence among Children? A Marginal Structural Modeling Analysis, Southwest Ethiopia

**DOI:** 10.4314/ejhs.v30i5.3

**Published:** 2020-09

**Authors:** Firew Tiruneh, Yared Deyas

**Affiliations:** 1 Department of Midwifery, Collage of Health Science, Mizan Tepi University, Mizan Teferi, Ethiopia; 2 Department of Midwifery, Collage of Health Science, Mizan Tepi University, Mizan Teferi, Ethiopia

**Keywords:** Marginal structural model, children, HAART, TB incidence

## Abstract

**Background:**

Children younger than 15 years, carry almost 80% of the global burden of HIV/AIDS. HIV worsens the progression of latent TB to active TB disease. Although antiretroviral treatment has shown marked reduction in Tuberculosis incidence, TB continues to occur in Sub-Saharan countries including Ethiopia. The aim of this study was to investigate the impact of HAART on the incidence of tuberculosis among children infected with HIV in Southwest Ethiopia.

**Methods:**

A retrospective cohort study was conducted between 2009 to 2014. We used chi-square test, and Mann-Whitney U test to compare non-HAART and HAART cohort. We estimated the effect of HAART on TB incidence using marginal structural model after adjusting for time-dependent confounders affected by exposure.

**Result:**

A total of 844 children were followed. We observed them for a median of 51 months (IQR 31) and a total of 2942.99 child-years. The overall TB incidence rate was 7.917 per 100 child years (95% CI, 6.933–9.002). TB incidence for specific HAART and non-HAART cohort were 7.67 per 100 child-years (95% CI, 6.318–9.217) and 8.17 per 100 child-years (95% CI, 6.772–9.767) respectively. From marginal structural modeling, children on HAART were 36% (HR=0.642, 95% CI 0.442–0.931, p<0.02) less likely to develop TB compared to those who were not.

**Conclusion:**

HAART reduced the hazard of TB in HIV-infected children by 36%. This is by far less than what is expected.

## Introduction

Children younger than 15 years, carry almost 80% of the global burden of HIV/AIDS. Infection with HIV is an important risk factor for Tuberculosis (1). Worldwide TB is the most common opportunistic illness and the leading cause of death among HIV infected children (2,3). A total of 1.5 million people died from TB in 2018 (including 251,000 people with HIV). In the same year, 1.1 million children fell ill with TB globally, and there were 20% child deaths due to TB (including among children with HIV)(4). According to WHO, there were an estimated 0.9 million new cases of TB amongst people who were HIV-positive, 72% of whom were living in Africa (3). Around 15–20% of TB cases were estimated among children in settings with a high burden of TB (5,6).

HIV and TB form a lethal combination, each speeding the other's progress (7,8). People with Latent TB have lifetime risk of 10–15% to develop active TB disease. The risk of developing TB in HIV-infected children is 20 times higher than HIV-uninfected children (9). Without treatment, 15–50% of HIV-positive infants and children will develop active TB within two years after becoming infected with TB (10).

To prevent TB, highly active antiretroviral therapy(HAART) is one of the strategies recommended by the World Health Organization (WHO) (11,12). HAART has shown marked reduction in the incidence of Tuberculosis (13–15). Although the risk of developing TB is reduced by 70%–90% among HIV-infected persons receiving HAART compared with untreated individuals (16), TB risk remains high following HAART initiation (17,18).

Higher risk may be partially due to incomplete immune restoration and “unmasking” of previously undiagnosed TB in the setting of immune reconstitution (19). The effect of HAART on TB incidence in resource constrained country may be adversely affected by multiple factors including delayed presentation to care and a higher incidence of co-occurring infectious and non-infectious conditions such as under nutrition and adherence level (13).

HIV-infected children are most at risk to develop tuberculosis. Globally, from estimated 10 million new TB infections, 1 million of them were children less than 15 years of age in 2017 (20). In Ethiopia, reported number of PLHIV treated with HAART were 71%, and out of this 32% have suppressed viral load measurement in 2017 (21). TB case notification for TB was 6% with rapid diagnostic tool while TB preventive treatment reached 47% (22). Routine screening and treatment for latent TB infection (LTBI) has been shown to reduce the risk of active TB in HIV-infected patients (23).

In Ethiopia, few studies examined the incidence of TB among children on HAART. These few studies have attempted to draw a causal inference regarding the effect of HAART on TB(6,24,25). However, their analysis was biased. The bias comes from using standard methods for estimating treatment effect in cohort study. Standard method cannot allow us control time-varying confounders. The bias in estimating the causal effect of treatment in the presence of time-varying confounders could be addressed using marginal structural modeling: a method based on deriving inverse-probability-of-treatment-weights, which is then used in a pooled logistic regression model (26). We estimated effect of HAART on the incidence of TB among HIV infected children using marginal structural regression modeling after adjusting and weighting baseline and time varying covariates.

## Materials and Methods

**Study design and setting**: A retrospective cohort study was conducted for five years from 2009 to 2014. The study was conducted in selected ART clinics found in southwest Ethiopia. Southwest Ethiopia encompasses five zones; namely, Jimma, Illu Ababora, Kafa, Sheka and Bench-Maji.

The following definitions of terms were used in this study.

**Tuberculosis case**: (1) Smear positive result of acid-fast bacilli or (2) TB confirmed by culture or (3) clinical sign and symptoms compatible with TB infection criteria by WHO or (4) Chest radiography confirmation.

**Children on HAART**: Those who took combination therapy of 3 antiretroviral drugs that included two non-nucleoside reverse transcriptase and one protease inhibitor.

**Censored**: Children who were lost, died, or transferred out or did not develop the events until the last visit.

**Source and study population**: All children younger than 15 years having a follow-up care in ART clinics in southwest Ethiopia were source population. Study population were all randomly selected HIV positive under 15 years old children registered from September 2009 to August 2014 in Southwest Ethiopia.

**Exposure**: Treatment with highly active antiretroviral treatment (HAART) for at least 2 months.

**Outcome**: TB illness.

**Inclusion and exclusion criteria**: All under 15 years old children on HAART or pre-HAART follow-up who were registered from September 2009 to August 2014 were included in the study. However, all under 15 years old children who started anti-TB treatment at the beginning of follow-up and/or diagnosed as TB patients were excluded.

**Sample size and sampling procedure**: Total, 844 samples were determined using epi info™ Version 7. We took case-to-control ratio of 1:1.

Fresh list of pediatrics ART Clinic in each zone were prepared. Then with proportional allocation methods, samples were selected from each ART clinic via systematic random sampling technique for both ART and pre-ART cohort.

**Data collection tool and procedure**: A standardized tool which was adapted from existing literature was used. Adapted tools were translated into local languages by language experts. Then, relevant data was collected from patients' pre-ART and ART follow-up log books, database and other clinical records. To assure quality of data extraction, a pretest was conducted on 5% of the sampled population.

**Data entry and analysis**: The collected data were coded, and double entry was made in EpiData version 3.1 statistical package. The analysis was made by Statistical Package for the Social Sciences (SPSS) version 23 software. Before analysis, the data were processed and cleaned by running frequency, sorting and cross-tabulation. Descriptive statistical techniques were carried out to describe the characteristics of the study subjects.

We compared proportion of TB between non-HAART and HAART cohorts by chi-square test. Besides, we compared median time of TB incidence between non-HAART and HAART cohorts using the Mann-Whitney test because the populations were independent and not normally distributed. The incidence rate was calculated with Open Epi software and were expressed per 100 child-years of follow-up on HAART. The TB free survival probability were constructed by the Kaplan-Meier method and compared between non-HAART and HAART groups by the log-rank test. The comparison was two- tailed with p-values < 0.05.

We estimated the effect of HAART on TB incidence by adjusting for confounders measured at baseline and time-varying intermediates. We did this by fitting weighted pooled logistic regression model to construct stabilized inverse probability of treatment and censoring (IPTC) weights. Then, these IPTC weights were used in a weighted pooled logistic regression model to approximate the parameters of a marginal structural Cox model (26). Confounders were selected based on previous studies. The covariates included in these models were age, sex, baseline clinical stage and baseline CD4 as baseline covariates. The time varying covariates were CD4, Clinical stage, follow-up status, adherence and CPT.

Bivariable and multivariable Cox proportional hazard regression models were used to see independent predictors of TB incidence. Variables with p-value <0.2 in bivariable analysis were transferred to Multivariable cox proportional hazard model. In Multivariable analysis, variables with P-value <0.05 at 95% confidence level were considered as statistically significant predictors of TB incidence. The results were expressed as hazard ratios (HRs) with 95% confidence intervals (CI).

## Results

Of the total sample (844), 94.8% children were retrospectively followed. Half of them (50%) were non-HAART while 50% were HAART initiated. The median follow-up period for non- HAART was 47 months with IQR of 31 months whereas HAART initiated were followed for a median of 53 months with IQR of 32 months. The total follow-up period for non- HAART children and HAART initiated were 1469.08 and 1473.91 child years time respectively. The median and inter quintile range (IQR) of age for HAART cohort were 9 and 6 years respectively. The corresponding values for the pre-ART cohort were 7 and 5 years respectively. Regarding gender, males constitute the largest proportion in both HAART (51%) and non-HAART (56.8%) groups.

During a total of 2942.99 child-years followup, 506 OIs occurred in 248 children (31%). The most common reported OIs were pneumonia (22%) and TB (29.1 %). Nearly 2% children died, 7.8% transferred out, and 12.3% were lost to follow-up. Of 12.3% lost to follow-up, 35.7% were females, and the rest 64.3% were males. Sixty-five percent of the study subjects were either in WHO clinical stage I or II, while 65.3% had CD4 count above the threshold at enrollment.

Baseline characteristics, children on HAART compared to those non-HAART later had a degree of advanced WHO clinical stage (p< 0.001). Children who had a baseline CD4 count below threshold had a more advanced HIV clinical stage (p=0.03), and they were more likely to have Tuberculosis (p<0.01). Children who initiated HAART had no difference compared to those who did not regarding gender (p=0.10). However, there was a difference regarding the medians of age across categories of the HAART cohort (p<0.001). The characteristics of study subjects are indicated in Table 1.

**Table 1:** Characteristics of HAART initiated and HAART naïve children

Time period	Characteristics	Category	HAART naïve		HAART initiated	
			TB(No)	TB (yes)	TB(No)	TB (yes)
Baseline	Age	<1	3(27.3%)	8(72.7%)	0(0%)	18(100%)
		1–3	56(59.6%)	38(40.4%)	21(48.8%)	22(51.2%)
		3–5	45(81.8%)	10(18.2%)	41(67.2%)	20(32.8%)
		5–15	176(73.3%)	64(26.7%)	225(80.9%)	53(19.1%)
	Sex	Male	148(65.2%)	79(34.8%)	127(62.3%	77(37.7%)
		Female	132(76.3%)	41(23.7%)	160(81.6%)	36(18.4%)
	WHO clinical stage	I	158(75.2%)	52(24.8%)	152(78.8%)	41(21.2%)
		II	106(74.1%)	37(25.9%)	95(70.9%)	39(29.1%)
		III	16(34.0%)	31(66.0%)	34(57.6%)	25(42.4%)
		Iv	0(0%)	0(0%)	6(42.9%)	8(57.1%)
	CD4	Below threshold	73(62.9%)	43(37.1%)	103(65.2%)	55(34.8%)
		Above threshold	207(72.9%)	77(27.1%)	184(76.0%)	58(24.0%)

*WHO-world health organization, ‡TB-Tuberculosis

During the 2942.99 child-years of follow-up, 189 children developed TB. The overall TB incidence rate was 7.917 per 100 child years (95% CI, 6.933–9.002, whereas among HAART 7.667 per 100 child-years (95% CI, 6.318–9.217) and 8.1686 per 100 child-years (95% CI 6.772–9.767) for HAART naïve. The medians of time to event observation for children who started HAART was statistically similar with the medians observed for untreated children (p=0.229). In comparison, the proportion of non-HAART children who developed TB (29.5%) were higher than the 17.8% of HAART-initiated. However, no significant statistical difference was observed in terms of TB incidence between children on HAART and non-HAART (p=0.641).

The risk of TB was higher among children whose CD4 cell count was below threshold (200 cells/mm^3^) compared to those children having CD4 count above threshold (200 cells/mm^3^). The incidence of TB had a direct relationship with ageas the age increased the risk also increased (p<0.001).

An unadjusted model that did not account for any confounder estimated no protective effect of HAART on TB incidence, relative to HAART naïve (HR 1.019, 95% CI 0.788–1.318, P=0.885). The unweighted model that adjusted baseline confounder suggested (HR 0.933, 95% CI 0.98-0.712-1.224, P=0.618). Similarly, when we fitted weighted model appropriately accounting for baseline confounder, the result did not suggest protective effect of HAART (HR, 0.903, 95% CI: 0.657–1.240, P=0.529). However, the model that accounted for both the baseline and time-varying confounders using marginal structural models showed that HAART reduces the risk of tuberculosis; the HR from IPTC-weighted model was (HR, 0.642, 95%CI, 0.442–0.931, P=0.020) Table 2.

**Table 2:** Estimated effect of HAART on TB incidence among 800 HIV-infected children initiating HAART, southwest Ethiopia, 2010–2014

Model	HR	95% CI	p-value
**Unweighted and unadjusted**	1.019	0.788–1.318	0.885
**Unweighted but adjusted**	0.933	0.712–1.224	0.618
**Baseline Weighted and stabilized**	0.903	0.657–1.240	0.529
**Time-varying Weighted and stabilized**	0.683	0.490–0.952	0.024
**Total Weighted and stabilized**	0.642	0.442–0.931	0.020

## Discussion

The finding of this study showed, HAART noticeably had a protective effect against Tuberculosis among HIV infected children. Using a marginal structural model, we estimated that HAART reduced the rate of TB incidence by 36% relative to non-HAART. Although our method of analysis was different, this finding was supported by a study that estimated TB incidence among children after HAART was initiated (27–29). Our finding was in line with the effect observed with similar methods of analysis among adults in Europe and the United States (30). The similarity of these findings offers evidence that HAART is effective against TB in HIV-infected children in Southwest Ethiopia.

The effect of HAART estimated in our study was lower than the effect revealed by similar studies in high income countries (31,32). This difference could be resulted from the fact that pediatric HIV programs in Southwest Ethiopia like sub-Saharan Africa, often face challenges that could adversely increase TB among HIV infected children, including limited diagnostic capacity, delayed healthcare seeking and poor retention in care and under-nutrition (33). These factors underline why a similar protective effect of HAART on TB in children could not be expected across different settings.

The incidence of tuberculosis among children on HAART in this study was 7.7 per 100 child-years and for non-HAART group was 8.2 per 100 child-years. Mathematically, it seems low; however, there is no statistically significant difference between the two groups. This finding is different from studies that supported reduced incidence of tuberculosis among HAART initiated (14,34,35).

On the other hand, this study revealed lower incidence of TB among children on HAART compared to studies conducted in different parts of Ethiopia (6,24,25). The reasons for lower incidence rate in this study could be related to difference in study setting and period. Besides, lower incidence rate of TB could be related to limited access to health services and poor screening practice. The lower incidence can also be attributed to incompleteness of data on some patients which resulted in under estimation of incidence.

In this study, high TB incidence was observed among CD4+ count strata below threshold for age during follow-up period. The result depicted an opposite relationship between duration on HAART and TB incidence. This finding is consistent with other studies, and this may be due to better TB-specific immune repair with time spent on HAART (36)(37). We demonstrated that the high TB incidence rates observed in this study may be due to high ongoing community level TB transmission. Poor knowledge of the community regarding TB and poor health care access may resulted in higher TB incidence. Most importantly, this might be justified with our selection criteria. We selected cases with all diagnostic criteria including clinical criteria. The criteria might be less specific but highly sensitive.

Our study has some limitations. In our selection criteria we suspected that we imposed unintentional classification of prevalent tuberculosis as incident tuberculosis. Children who received no screening for tuberculosis in their enrolment were followed and if in the meantime TB was developed then it was considered as incident cases. Besides, HAART was not randomly assigned, and thus the possibility of residual confounding cannot be ruled out. We also acknowledge that using clinical TB diagnostic criterion was the limitation of our study.

The effect of HAART on TB incidence among HIV infected children in this study is the first valid estimate, to our knowledge, of the extent to which therapy decreases TB incidence in a highly relevant population of HIV-infected children living in Southwest Ethiopia. We analyzed data using marginal structural modeling to establish causal inference of HAART on TB incidence, which would be mentioned as strength of the study. Tuberculosis incidence in the study area was relatively high enough to be a public health problem.
